# Genetic diversity of *Staphylococcus aureus* wall teichoic acid glycosyltransferases affects immune recognition

**DOI:** 10.1099/mgen.0.000902

**Published:** 2022-12-07

**Authors:** Sara M. Tamminga, Simon L. Völpel, Kim Schipper, Thilo Stehle, Yvonne Pannekoek, Nina M. van Sorge

**Affiliations:** ^1^​ Department of Medical Microbiology and Infection Prevention, Amsterdam UMC, University of Amsterdam, Amsterdam, The Netherlands; ^2^​ Interfaculty Institute of Biochemistry, University of Tübingen, Tübingen, Germany; ^3^​ Department of Pediatrics, Vanderbilt University School of Medicine, Nashville, TN, USA; ^4^​ Netherlands Reference Laboratory for Bacterial Meningitis, Amsterdam UMC, Amsterdam, The Netherlands

**Keywords:** glycosylation, PubMLST, *Staphylococcus aureus*, *tar *glycosyltransferases, wall teichoic acid

## Abstract

*
Staphylococcus aureus* is a leading cause of skin and soft tissue infections and systemic infections. Wall teichoic acids (WTAs) are cell wall-anchored glycopolymers that are important for *S. aureus* nasal colonization, phage-mediated horizontal gene transfer, and antibiotic resistance. WTAs consist of a polymerized ribitol phosphate (RboP) chain that can be glycosylated with *N*-acetylglucosamine (GlcNAc) by three glycosyltransferases: TarS, TarM, and TarP. TarS and TarP modify WTA with β-linked GlcNAc at the C-4 (β1,4-GlcNAc) and the C-3 position (β1,3-GlcNAc) of the RboP subunit, respectively, whereas TarM modifies WTA with α-linked GlcNAc at the C-4 position (α1,4-GlcNAc). Importantly, these WTA glycosylation patterns impact immune recognition and clearance of *S. aureus *. Previous studies suggest that *tarS* is near-universally present within the *S. aureus* population, whereas a smaller proportion co-contain either *tarM* or *tarP*. To gain more insight into the presence and genetic variation of *tarS*, *tarM* and *tarP* in the *S. aureus* population, we analysed a collection of 25 652 *S *. *aureus* genomes within the PubMLST database. Over 99 % of isolates contained *tarS*. Co-presence of *tarS*/*tarM* or *tarS*/*tarP* occurred in 37 and 7 % of isolates, respectively, and was associated with specific *S. aureus* clonal complexes. We also identified 26 isolates (0.1 %) that contained all three glycosyltransferase genes. At sequence level, we identified *tar* alleles with amino acid substitutions in critical enzymatic residues or with premature stop codons. Several *tar* variants were expressed in a *S. aureus tar*-negative strain. Analysis using specific monoclonal antibodies and human langerin showed that WTA glycosylation was severely attenuated or absent. Overall, our data provide a broad overview of the genetic diversity of the three WTA glycosyltransferases in the *S. aureus* population and the functional consequences for immune recognition.

## Data Summary

All data used for analysis are available as open source data at PubMLST *S. aureus* BIGSdb (https://pubmlst.org/organisms/staphylococcus-aureus).

**Impact Statement**

Wall teichoic acids (WTAs) are cell wall-anchored glycopolymers of *Staphylococcus aureus* that consist of a polymerized ribitol phosphate (RboP) chain that can be glycosylated with *N*-acetylglucosamine (GlcNAc) by three glycosyltransferases: *TarS*, *TarM,* and *TarP*. Each glycosyltransferase modifies WTA differently, resulting in different WTA glycosylation patterns that impact immune recognition and clearance of *S. aureus *. To gain more insight into the presence and genetic variation of *tarS*, *tarM*, and *tarP* in the *S. aureus* population, we analysed a collection of 25 652 *S. aureus* genomes within the PubMLST database. We found that over 99 % of isolates contained *tarS*. Co-presence of *tarS*/*tarM* or *tarS*/*tarP* occurred in 37 and 7 % of isolates, respectively, and was associated with specific *S. aureus* clonal complexes. At sequence level, we identified tar alleles with amino acid substitutions in critical enzymatic residues or with premature stop codons. Expressing these *tar* variants in a *S. aureus tar*-negative strain showed that WTA glycosylation was severely attenuated or absent, emphasizing that gene absence or presence does not always predict phenotype. Studying the genetic presence and diversity of *tarS*, *tarM,* and *tarP* provides more insight into *S. aureus* WTA glycosylation, which can help in the development of anti-* S. aureus* preventive or therapeutic interventions such as monoclonal antibodies, phage therapy and vaccines. As such, our study provides a blueprint to dissect and functionally analyse *S. aureus* genes at a population-wide level.

## Introduction

*Staphylococcus aureus* is a common member within microbiota communities and colonizes approximately 30 % of the human population asymptomatically [1]. However, *S. aureus* is also a prominent bacterial pathogen in hospital- and community-acquired infections. Infections often start locally, for example in the skin, but if local immune recognition and immune defence fail, bacteria can disseminate and cause systemic infections. Even with timely treatment and clinical management, such infections are associated with high overall disease burden and mortality.

A major component of the *S. aureus* cell wall is wall teichoic acid (WTA), which is important for nasal colonization, β-lactam resistance and phage-mediated horizontal gene transfer [2, 3]. WTA molecules are composed of 20–40 ribitol phosphate (RboP) subunits that are polymerized into a linear backbone, which is modified with d-alanine and *N*-acetylglucosamine (GlcNAc) moieties [4]. Currently, three different glycosyltransferases, i.e. TarS, TarM and TarP, are known to glycosylate WTA with GlcNAc [4–9]. GlcNAc is attached to the C-4 hydroxyl group of RboP in either the α- or β-configuration by TarM and TarS, respectively [4, 9]. Similar to TarS, TarP attaches GlcNAc in a β-configuration, but to the C-3 hydroxyl group of RboP [7]. All three enzymes have been characterized both functionally and structurally, providing insight into critical protein residues and features for catalysis [5–8].

The specific WTA GlcNAc modifications greatly influence β-lactam- and phage resistance of *S. aureus* [2, 4] as well as host–pathogen interactions [10–13]. For instance, the WTA glycan modifications represent dominant antigens in the *S. aureus *-reactive antibody pool in humans [2, 10, 13]. Most antibodies are directed against β-GlcNAc-modified WTA [10] and not every WTA glycoform may be similar in terms of immunogenicity or as a target for antibody-mediated clearance [7, 10]. With regard to innate immunity, β-GlcNAc-WTA but not α-GlcNAc-WTA is detected by the innate receptor langerin, which is expressed on skin epidermal Langerhans cells (LCs) [11, 12]. These findings indicate that different WTA glycosylation profiles, which depend on the presence and activity of the three known glycosyltransferases, impact *S. aureus* immune recognition and clearance.

Previous studies suggest that *S. aureus* near-universally contains *tarS,* whereas a smaller proportion co-contains either *tarM* or *tarP* [1, 7, 14]. Indeed, *tarS* is part of the *S. aureus* core genome and clusters with well-studied WTA biosynthesis genes [4]. In contrast, *tarM* is located elsewhere in the genome and is suggested to be an ancient genetic trait of *S. aureus *, based on the observation that strains belonging to the very early branching *S. aureus* bear both *tarS* and *tarM* in their genome [2, 14]. It is hypothesized that *tarM* was lost during *S. aureus* evolution, resulting in its absence in strains belonging to clonal complex (CC) 5 and CC398 [14]. The most recently identified enzyme, *tarP*, is encoded on different prophages and its presence seems to be restricted to isolates belonging to CC5 and CC398 [7]. However, an overview on the presence, co-presence and genetic variation of *tarS*, *tarM* and *tarP* within the *S. aureus* population is currently lacking.

In this study, we dissected the presence and genetic variation of *tarS*, *tarM* and *tarP* in a collection of 25 652 *S *. *aureus* genomes that are deposited in the open-access PubMLST database (https://pubmlst.org/organisms/staphylococcus-aureus). We also analysed whether specific combinations or *tar* allelic variants were associated with specific CCs of *S. aureus *. Finally, we performed *in silico* analyses followed by cloning and plasmid-expression of several *tar* variants in a *S. aureus tar*-deficient strain to observe the functional effect of *tar* sequence variation on WTA glycosylation using WTA-specific Fab fragments and human langerin. Overall, our data provide more insight into the genetic diversity of the three WTA GlcNAc-transferases and demonstrates how this natural variation can impact *S. aureus* immune recognition by both the innate and adaptive immune system. As such, our study provides a blueprint to dissect and functionally analyse *S. aureus* genes at a population-wide level.

### Methods

### *S. aureus* PubMLST database analysis

We analysed the *S. aureus* genomes deposited in the PubMLST Bacterial Isolate Genome Sequence Database (BIGSdb) [15] to determine the presence and genetic diversity of *tar*-glycosyltransferases *tarS*, *tarM* and *tarP* (identified as SAUR2940, SAUR2942 and SAUR2941, respectively in the PubMLST *S. aureus* database). Of the 26 605 genomes in the database (extracted on 8 February 2022), several genomes were excluded from further analysis based on the following criteria. Fifty-seven isolates were excluded since they were suspected to be contaminated or not *S. aureus,* based on the comments section of the database. Furthermore, 691 isolates were excluded because they contained more than 300 contigs and/or had a N50 contig length of <20 000 bp. Strains were also excluded when a *tar*-gene was not present within a single contig (*n*=205). After the exclusion of these strains, 25 652 *S *. *aureus* isolates remained for analysis. Allele numbers and nucleotide sequences of the three glycosyltransferases from these isolates were downloaded from the PubMLST *S. aureus* BIGSdb [15]. To verify that all isolates that contained a *tar-*gene also had an assigned allele in the database, an additional blast analysis was performed using allele 1 for each of the *tar* genes and default settings as defined in PubMLST (blastn word size of 11; blastn scoring parameters: reward: 2; penalty: −3; gap open: 5; gap extend: 2) [15]. Isolates that contained a *tar*-gene sequence but lacked an assigned allele were scanned using default parameters as defined in PubMLST (minimal nucleotide identity of 70 %; minimal alignment of 50 % of allele sequence; blastn word size of 20) [15]. New alleles were assigned and submitted to the database. In the case of a premature stop codon in a *tar* sequence, the gene was marked as ‘truncated’, and no allele was assigned. Information on the isolates’ sequence type (ST) and CC was also obtained from the PubMLST *S. aureus* BIGSdb. For some isolates the CC in the database was not defined, and therefore we manually added CC7, CC12, CC25, CC59, CC88, CC130, CC133, CC398 and CC425 based on ST information [16, 17].

### Alignments and protein sequence analysis

The nucleotide sequences of all allelic variants of *tarS*, *tarM* and *tarP* were translated to amino acid sequences and aligned using muscle in mega version 11 [18]. We manually analysed critical residues of the enzymes (based on previous studies) [5–8] for amino acid substitutions in or ±1 amino acid next to the critical residues of Tar-enzymes. The effect of the amino acid substitutions on interactions with the acceptor substrate (poly-RboP) or the donor substrate (UDP-GlcNAc) was predicted and visualized using the PyMOL Molecular Graphics System, Version 2.5.1 (Schrödinger). During visualization, we continuously chose the rotamer orientation for the mutated residue that had the fewest strains and clashes with other residues in the structure.

### Bacterial strains and culture conditions

All plasmids and *S. aureus* strains used for wet-lab experiments in this study are listed in Table S1 (available in the online version of this article). Bacteria were grown overnight in 5 ml tryptic soy broth (TSB; Oxoid) at 37 °C with agitation. For *S. aureus* strains that were complemented with plasmid, TSB was supplemented with 10 µg ml^−1^ chloramphenicol (Sigma). Overnight cultures were subcultured the next day in fresh TSB and grown to mid-exponential growth phase, corresponding to an optical density of 0.6–0.7 at 600 nm (OD_600_).

### Generation of complemented RN4220 Δ*tarM*S strains with *tar* variants

Shuttle vector RB474 [19] containing full-length copies of *tarS, tarM* or *tarP* as inserts (Table S2) were used to recreate naturally occurring amino acid substitutions and premature stop codons in *tar*-glycosyltransferases [4, 7, 9]. Nucleotide substitutions were generated using either a QuikChange Site-Directed Mutagenesis Kit (Agilent) or the required nucleotide sequence was ordered as gBlock (IDT). Used primers are listed in Table S3. Plasmids containing sequences of *tarS*, *tarM* or *tarP* were amplified in *Escherichia coli* DC10b [20] or *E. coli* XL-1 and transformed into electrocompetent *S. aureus* RN4220 Δ*tarMS* [3] through electroporation with a Bio-Rad Gene Pulser II (2.0 kV, 600 Ω, 10 µF). After recovery, bacteria were plated on tryptic soy agar (TSA) plates supplemented with 10 µg ml^−1^ chloramphenicol to select plasmid-complemented colonies. The presence of *tarS, tarM* or *tarP* was confirmed by PCR analysis using *tar* gene-specific primers, and nucleotide sequences of the constructs were confirmed by Sanger sequencing.

### Analysis of WTA glycosylation using monoclonal Fab fragments and human langerin-FITC

The enzymatic activity of the *tar-*variants was assessed by analysing WTA glycosylation using WTA-specific Fab fragments [12]. Bacteria were grown to mid-exponential growth phase, collected by centrifugation (4000 rpm, 8 min, 4 °C) and resuspended at an OD_600_ of 0.4 (∼1×10^8^ c.f.u. ml^–1^) in PBS (pH 7) with 0.1 % BSA (Sigma). Bacteria (1.25×10^6^ c.f.u.) were incubated with 3.3 µg ml^−1^ monoclonal Fab fragments (pre-diluted in PBS 0.1 % BSA) specific to β-GlcNAc (clone 4497) or α−1,4-GlcNAc (clone 4461) WTA, respectively [12]. After washing, bacteria were incubated with a goat F(ab′)_2_ anti-human Kappa-Alexa Fluor 647 (pre-diluted in PBS 0.1 % BSA; 2.5 µg ml^−1^, Southern Biotech #2062–31) to detect bound Fab fragments. Bacteria were washed and fixed in PBS with 1 % paraformaldehyde, and analysed by flow cytometry on a BD FACSCanto II Flow Cytometer (BD Bioscience). Per sample, 10 000 gated events were collected and fluorescence was expressed as the geometric mean. Additionally, bacterial binding to recombinant FITC-labelled human langerin (kindly provided by Prof. C. Rademacher, University of Vienna, Vienna, Austria) was assessed as previously described [11]. Briefly, bacteria were grown to mid-exponential phase, collected and resuspended at an OD_600_ of 0.4 in TSM buffer [2.4 gl^–1^ Tris (Sigma-Aldrich), 8.77 gl^–1^ NaCl (Merck), 294 mg l^−1^ CaCl_2_(H_2_O)_2_ (Merck), 294 mg l^−1^ MgCl_2_(H_2_O)_6_ (Merck), containing 0.1 % BSA (Sigma), pH 7]. Next, bacteria were incubated with shaking for 30 min at 37 °C with 20 µg ml^−1^ FITC-labelled human langerin-extracellular domain (ECD) constructs (referred to as langerin-FITC). Finally, bacteria were washed once with TSM 0.1 % BSA, fixed in 1 % paraformaldehyde in PBS and analysed by flow cytometry as described above.

### Statistical analysis

Flow cytometry data were analysed using FlowJo 10 (FlowJo). Data were analysed using GraphPad Prism 9.1.0 (GraphPad Software) with a Chi-Square Goodness of Fit test or a one-way ANOVA followed by a Dunnett’s multiple comparison test. The *P*-values are depicted in the figures or mentioned in the caption, and *P*<0.05 was considered significant.

## Results

### *TarS* is present in >99% of all *S. aureus* isolates

Analysis of 25 652 *S *. *aureus* genomes demonstrated that over 99 % of isolates contained *tarS*, with 36.7 and 6.6 % of isolates co-containing *tarM* (*n*=9 404) or *tarP* (*n*=1 702), respectively (Table 1). We also found 26 isolates (0.10 %) that contained all three glycosyltransferase genes (Table 1). Overall, to 186 isolates no *tarS* alleles could be assigned in the following combinations: to 67 isolates (0.26 %) no *tar* alleles could be assigned, to 58 isolates (0.23 %) only a *tarP* allele could be assigned*,* to 56 isolates (0.22 %) only a *tarM* allele could be assigned, and to five isolates (0.019 %) both *tarP* and *tarM* alleles could be assigned (Table 1). Further analysis of these strains lacking a *tarS* allele identified an incomplete ORF for *tarS* in 165 out of 186 isolates (88.7 %) due to the presence of a premature stop codon. We also found premature stop codons in *tarM* (*n*=106) and *tarP* (*n*=2) sequences. Isolates with premature stop codons in specific *tar* genes are indicated as ‘truncated’ in Table 1.

**Table 1.** Presence of *tar*S, *tarM* and *tarP* in 25652 *S. aureus* isolates

*tar* genotype	Isolates (*n*)		Percentage of total
*tarS*	14 334		55.9
*tarS* + truncated *tarM*		98	
*tarS* + truncated *tarP*		2	
*tarS* only		14 234	
*tarS* + *tarM*	9404		36.7
*tarS* + *tarP*	1702		6.6
*tarS* + *tarP* + truncated *tarM*		4	
*tarS* + *tarP*		1698	
No tar alleles assigned	67		0.26
truncated *tarS*		57	
truncated *tarM*		4	
No *tar* genes		6	
*tarP*	58		0.23
*tarP* + truncated *tarS*		58	
*tarM*	56		0.22
*tarM* + truncated *tarS*		45	
*tarM* only		11	
*tarS* + *tarM* + *tarP*	26		0.1
*tarM* + *tarP*	5		0.02
*tarP* + *tarM* + truncated *tarS*		5	
Total	25 652		100

### Presence of *tar*-glycosyltransferases is linked to *S. aureus* CCs

**Fig. 1.**
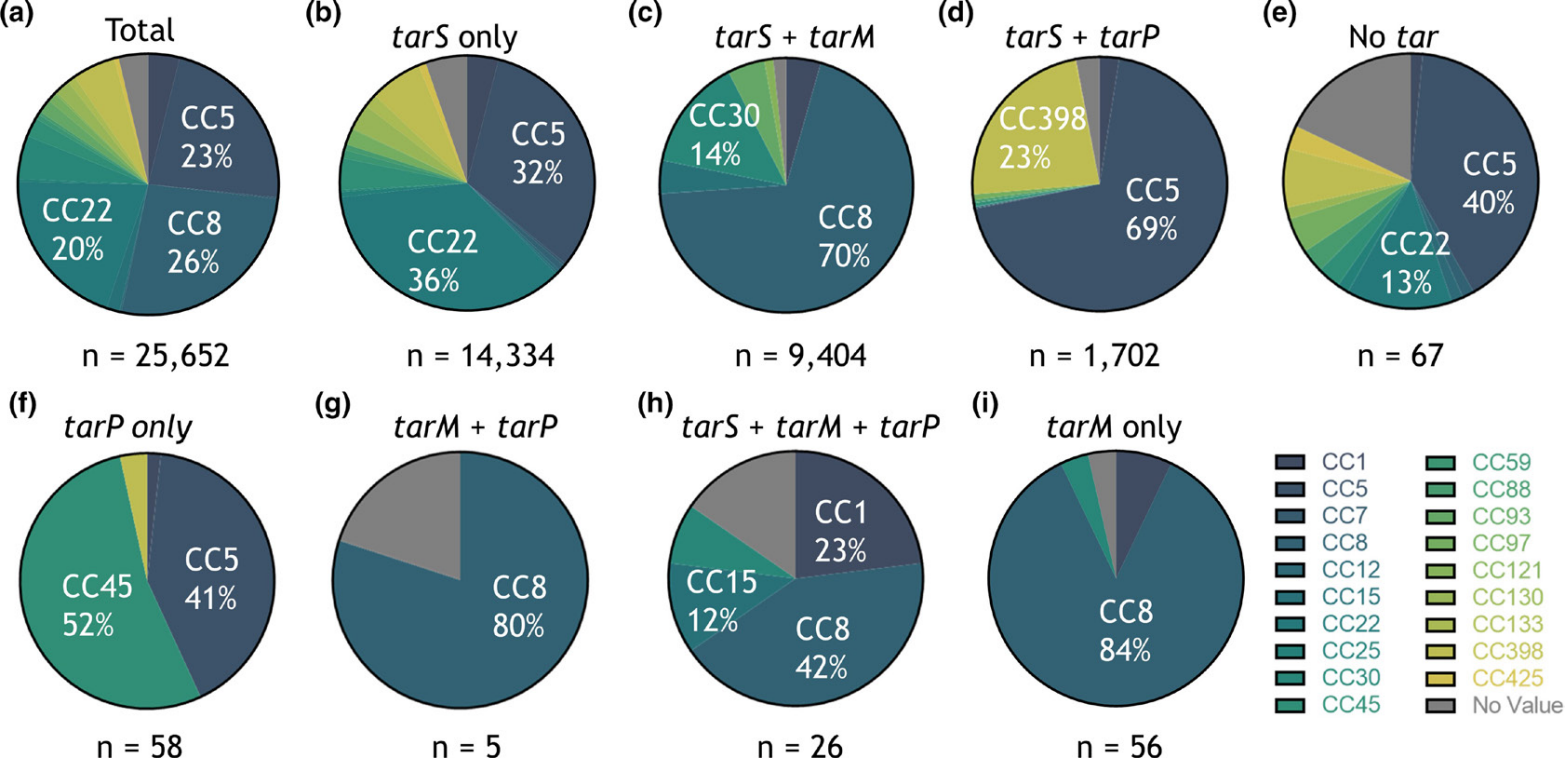
The *tar* genotype is linked to *S. aureus* clonal complexes. (**a**) Distribution of CCs for isolates within the PubMLST database on the extraction date. Only CCs that comprise >10 % of isolates are indicated. Colour coding of CCs is shown in the key on the right. (**b–i**) *S. aureus* CC distribution is shown for each *tar* genotype indicated as in (**a**). The difference between the CC distribution of each *tar* genotype compared to the total CC distribution was statistically significant for all *tar* genotypes (*P*<0.0001), except for (**g**) *tarM+tarP* (*P*=0.89).

Next, we analysed whether *tar* genotype was associated with specific CCs of *S. aureus *. The most prevalent CCs in the database were CC8 (26 %), CC5 (23 %) and CC22 (20 %) (Fig. 1a), which are all CCs associated with human disease and include methicillin-resistant *S. aureus* (MRSA) strains [17, 21–23]. Besides being a successful human pathogenic lineage, CC5 is also often found in poultry infections [16, 24]. Other livestock- and animal-associated CCs that were present in the database include CC97, CC130, CC133, CC398, and CC425 [16, 17, 22, 24]. Of the isolates that only contained a complete *tarS* gene, 36 % belonged to CC22 and 32 % to CC5 (Fig. 1b). Overall, the *tarS/tarM* combination was predominantly found in isolates belonging to CC8 (Fig. 1c), even when *tarP* (Fig. 1g, h) or a truncated version of the *tarS* gene was present (Fig. 1i). In total, *tarM* was found in seven different CCs (Table S4). For isolates that contain *tarS* and *tarP*, nearly all belonged to CC5 (69 %) or CC398 (23 %) as previously described [7] (Fig. 1d). Yet, *tarP* was also identified in at least 11 additional CCs (Table S4). Interestingly, of the 58 isolates that only contained a complete *tarP* gene, 52 % belonged to CC45 (Fig. 1f), whereas this CC represented only 0.4 % of the *tarS*/*tarP*-containing isolates.

### *Tar* alleles and CC distribution

**Fig. 2.**
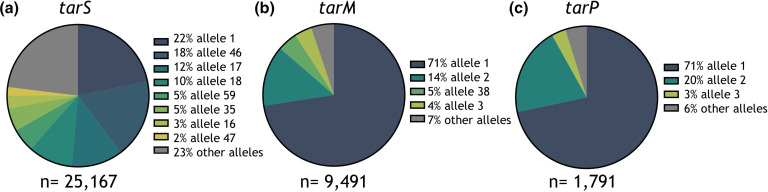
Allelic variants of *tarS, tarM* and *tarP* in the PubMLST *S. aureus* collection. (**a**) Representation of the dominant *tarS* alleles within the *S. aureus* isolates of the PubMLST database. Only alleles that cover >1 % of all sequences are displayed individually, while the remaining minor alleles are grouped (grey). The total number of alleles (based on nucleotide sequence) identified for *tarS* was 536. (**b**) Same as in (**a**) but for *tarM*; the total number of alleles was *n*=180. (**c**) Same as in (**a**) but for *tarP*; the total number of alleles was *n*=28.

Based on nucleotide sequence, 536 alleles (coding for 408 unique ORFs) were identified for *tarS* within the PubMLST *S. aureus* database, and eight alleles (coding for six unique ORFs) comprised 77 % of all *tarS* nucleotide sequences (Fig. 2a). The number of alleles was much smaller for *tarM* with 180 nucleotide sequences coding for 143 unique ORFs, and *tarP* with only 28 alleles coding for 19 unique ORFs. For these genes, allele 1 was most frequently found, representing 71 % of all *tarM*-sequences (Fig. 2b) and 71 % of all *tarP*-sequences (Fig. 2c). All CCs, except *tarS* CC1 and 8, were characterized by the presence of a single dominant allele of *tar-*glycosyltransferases (Fig. S1).

### Amino acid substitutions in critical residues of Tar-glycosyltransferases

**Fig. 3.**
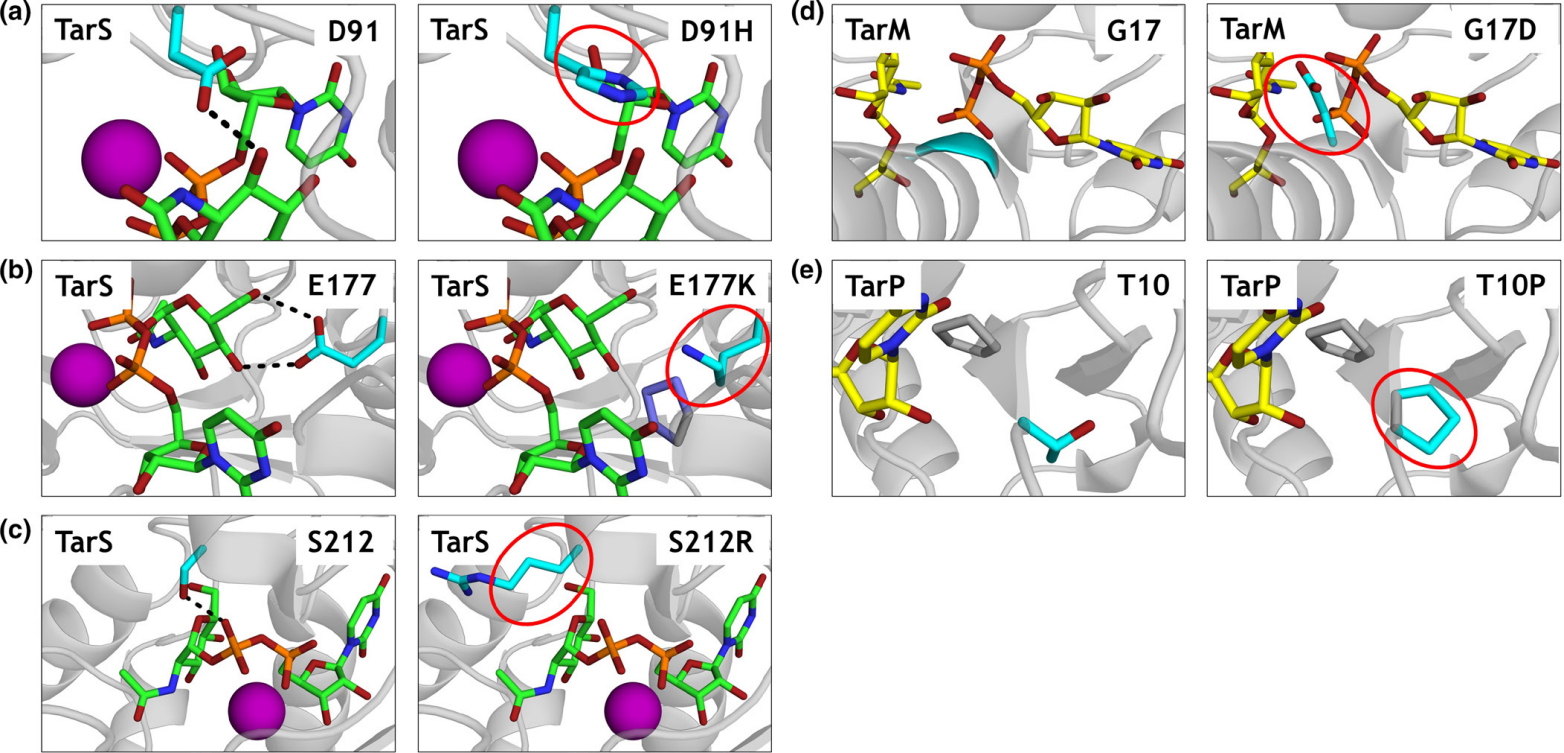
PyMOL visualizations of the effect of naturally occurring amino acid substitutions on molecular interactions within Tar-enzymes. (**a**) TarS is shown in grey cartoon presentation (PDB code 5TZE). The donor substrate UDP-GlcNAc (green) and the residues of interest: unaltered (left) and the substitution (right, circled red), both in cyan, are all displayed in stick form. Black dashed line indicates an interaction with UDP-GlcNAc. The Mn^2+^ ion coordinating the pyrophosphate of UDP is presented as a sphere in magenta. (**b**) In the E177K mutation (right) the conflicting residue P71 is displayed in stick form (slate). (**c**) TarS, S212 and S212R are displayed as in (**a**). (**d**) TarM is shown in cartoon presentation in grey (PDB code 4X7R). UDP and α-glyceryl-GlcNAc are displayed in stick form (yellow). The remaining residues are coloured as in (**a**). (**e**) TarP, T10 and T10P are shown as in (**d**) with P9 (grey) as a stick model (PDB code 6H4M).

We identified several *tar* alleles with a naturally occurring amino acid substitution in a critical residue of the enzyme, based on information from the published crystal structures [6–8]. Overall, these alleles were identified in 32 isolates (0.12 %) of the analysed *S. aureus* genomes. The amino acid changes in these variants and the function assigned to these residues are listed in Table 2. Fig. 3 visualizes the amino acid substitutions and their predicted effect on molecular interactions in PyMOL. D91 is part of the DxD motif in TarS, which is directly involved in the interaction with UDP-GlcNAc [6] (depicted as a black dashed line; Fig. 3a). Substitution of an acidic aspartate for a basic histidine, which also has a bulkier side chain, is predicted to affect UDP-GlcNAc binding (Fig. 3a). TarS E177 makes contact with the C4-OH and C6-OH of the UDP-GlcNAc moiety [6] (black dashed line; Fig. 3b) and supports orientation of D178 (base catalyst). Consequently, the substitution of E177 to K177 is likely to conflict with P71 (slate; Fig. 3b) in addition to a change from a positively to a negatively charged residue. Moreover, the distance of the terminal primary amine group to UDP-GlcNAc C4-OH and C6-OH becomes 4.5 Å (compared to 2.7 and 3.0 Å of the E177 carboxy group to UDP-GlcNAc C4-OH and C6-OH, respectively), which may hamper sufficient hydrogen bonding (Fig. 3b). A third mutation in *tarS* results in substitution of S212 to R212 (Fig. 3c). TarS S212 coordinates the β-phosphate of the donor substrate UDP-GlcNAc and is positioned in close proximity to both the donor and acceptor substrate binding sites [6]. During the amino acid substitution (S212R) visualization in PyMOL, we placed several rotamers that did not conflict with TarS structure (Fig. 3c). However, all these rotamers point to the spaces between the two sulphates (yellow; Fig. S2) which might indicate the phosphate binding sites of poly-RboP and therefore may interfere with binding of the acceptor substrate. Furthermore, the coordination of the β-phosphate might be lost as arginine is too long to facilitate this (Fig. 3c). For TarM, the backbone nitrogen atom of G17 coordinates the α-phosphate of UDP-GlcNAc [8]. Consequently, the substitution of G17 to D17 is predicted to affect the interaction of TarM with UDP-GlcNAc as there appears to be little space for the aspartate side chain (Fig. 3d). In TarP, no amino acid substitutions of critical residues were identified, but we did identify an amino acid substitution (T10P) next to a critical residue (F11). F11 stabilizes the uracil moiety of UDP with its aromatic side chain and interacts with the backbone carbonyl of the ribose moiety [7], and mutation of the neighbouring amino acid may affect this interaction (Fig. 3e). Moreover, T10P forms a proline–proline peptide bond with P9, and this diproline segment is even more restricted in its conformational arrangement compared to a single proline (Fig. 3e).

**Table 2.** Naturally occurring amino acid substitutions in *tarS*, *tarM* and *tarP* with function of the original residue

*tar* gene	Substitution	Isolates (*n*)	Function of the residue
*tarS*	D91H	14	Interaction with C3-OH of GlcNAc [6]
	E177K	6	Interaction with C4-OH and C6-OH of GlcNAc [6]
	S212R	3	Coordinates β-phosphate of UDP-GlcNAc [6]
*tarM*	G17D	2	Interaction with α-phosphate of the UDP-GlcNAc [8]
*tarP*	T10P	7	F11 is important for GlcNAc interaction [7]

### Premature stop codons found in *tar*-glycosyltransferase genes

**Fig. 4.**
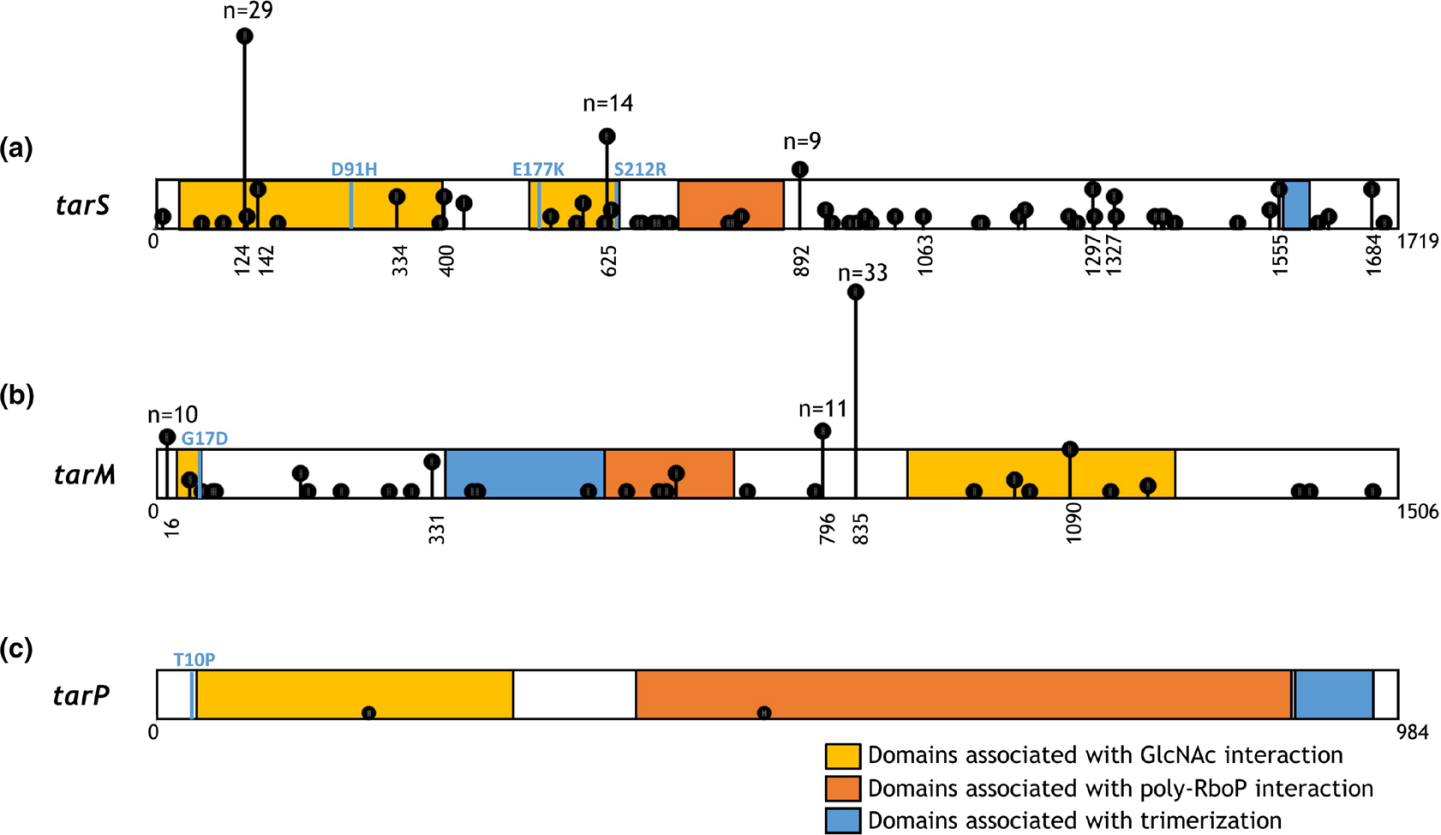
*Tar-*glycosyltransferase genes depicted as scaled-2D models with nucleotide position of premature stop codons. (**a**) Scaled 2D representation of *tarS* (nt 1719) containing two domains important for the GlcNAc interaction (nt 28–393 and 529–636; yellow), a poly-RboP interaction domain (nt 742–888; orange) and two residues associated with trimerization (nt 1561–1563 and 1594–1596; blue). Premature stop codons were identified in 165 isolates and are indicated by the vertical black lines that show position and frequency. For premature stop codons that were present in >4 isolates, the nucleotide position is shown, and for >8 the exact number of isolates is indicated. Amino acid substitutions are depicted in light blue. (**b**) *tarM* (1506 nt) contains two residues at the start (nt 49–54; yellow) and domain (nt 910–1233; yellow) that are important in the GlcNAc interaction. Domain 349–540 (blue) contains the HUB domain (formerly known as DUF1975) associated with TarM trimerization. Directly adjacent is the poly-RboP interaction domain (nt 543–699; orange). Premature stop codons (*n*=106 isolates) are indicated as in (**a**). (**c**) *tarP* (984 nt) contains a domain associated with the GlcNAc interaction (nt 31–285; yellow) followed by a poly-RboP interaction domain (385-789; orange). Domain 916–978 (blue) is associated with trimerization of the enzyme. Premature stop codons (*n*=2 isolates) are indicated as in (**a**).

Next, we wanted to predict the impact of the naturally occurring premature stop codons (indicated as truncated in Table 1). Therefore, we visualized the location of the stop codons in 2D-scaled models of *tar*-glycosyltransferases based on previously published enzyme structures [5–8] (Fig. 4)]. *TarS* (1719 nt) contains two domains that are important for GlcNAc interaction (nt 28–393 and 529–636; yellow) and a poly-RboP interaction domain (nt 742–888; red). Furthermore, TarS contains two residues that are associated with trimerization (nt 1561–1563 and 1594–1596; blue). Certain premature stop codon positions were found in five or more (up to 29) different isolates (Fig. 4a). A similar schematic overview is shown for *tarM* (Fig. 4b) and *tarP* (Fig. 4c). Premature stop codons in *tarM* (1506 nt) were identified in 106 isolates (Fig. 4b). For *tarP* (984 nt), only two isolates contained a premature stop codon (Fig. 4c). Overall, premature stop codons were found across the entire length of *tarS* and *tarM* genes and may affect enzymatic functionality depending on their position within the enzyme.

### Amino acid substitutions and premature stop codons hamper *S. aureus* immune recognition by antibodies and langerin

**Fig. 5.**
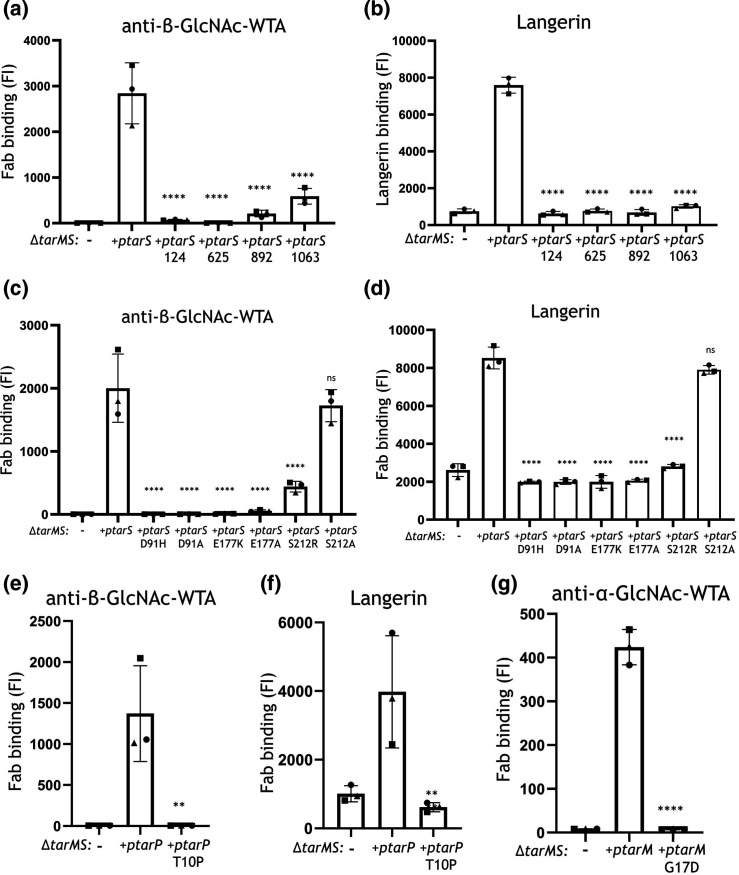
Impact of amino acid substitutions and premature stopcodons in TarS, TarM and TarP on *S. aureus* immune recognition. Binding of (**a, c, e**) monoclonal Fab fragments specific to β-GlcNAc-WTA (4497) and (**b, d, f**) human recombinant langerin-FITC to *S. aureus* RN4220 ∆*tarMS* complemented with plasmid-expressed WT *tarS* or premature stop codon *tarS* (**a, b**), *S. aureus* RN4220 ∆*tarMS* complemented with plasmid-expressed WT *tarS* or amino acid substitution *tarS* variants (**c, d**), or *S. aureus* RN4220 ∆*tarMS* complemented with plasmid-expressed WT *tarP* or amino acid substitution *tarP* variant (**e, f**). (**g**) Binding of Fab fragments specific to α-GlcNAc-WTA to *S. aureus* ∆*tarMS* complemented with plasmid-expressed WT *tarM* or amino acid substitution *tarM* variant. Data are depicted as geometric mean fluorescence intensity (FI) of three individually displayed biological replicates +standard deviation and were compared to plasmid-expressed WT enzyme: **P<0.01, ****P<0.0001.

The functional effect of naturally occurring amino acid substitutions and premature stop codons in *tar*-glycosyltransferases on WTA-GlcNAc decoration was assessed by expressing these *tarS, tarM* and *tarP* variants in a *S. aureus* mutant lacking WTA glycosylation (Δ*tarMS*). As a read-out of WTA glycosylation, we used specific Fab fragments against β-GlcNAc-WTA (clone 4497) and α−1,4-GlcNAc-WTA (clone 4461) [12]. Furthermore, we recently identified that β-GlcNAc-WTA is specifically detected by the human innate receptor langerin [11]. Therefore, we also determined the bacterial binding of the *tar* variants by langerin-FITC. For TarS, we tested the effect of a premature stop codon on nucleotide positions 124, 625, 892 and 1063 (Fig. 4a). Overall, β-GlcNAcylation of WTA is significantly (*P*<0.0001) decreased in all four premature stop codon variants of TarS compared to wild-type (WT) TarS but to varying extent. Premature stop codons on nucleotide positions 124 and 625 abrogated interaction with β-GlcNAc-specific Fab fragments and langerin (Fig. 5a, b). Closer to the C-terminus of TarS, stop codons at nucleotide positions 892 and 1063 severely hampered TarS function but still showed some residual activity, as β-GlcNAcylated WTA was still detectable with β-GlcNAc-specific Fab fragments (Fig. 5a). This level of glycosylation was insufficient for binding to langerin (Fig. 5b). Next, we analysed the effect of amino acid substitutions D91H, E177K and S212R in TarS and control mutations that were previously reported to attenuate TarS enzymatic activity *in vitro* [6]: D91A, E177A and S212A. Amino acid substitutions D91H and E177K in TarS completely abolished decoration of WTA with β-GlcNAc, similar to their controls D91A and E177A, as demonstrated by completely abrogated Fab binding (Fig. 5c, *P*<0.0001) and langerin binding (Fig. 5d, *P*<0.0001) compared to TarS WT (Fig. 5d). Of note, amino acid substitution S212R also significantly (*P*<0.0001) reduced WTA β-GlcNAcylation and thereby immune recognition (Fig. 5c, d). Interestingly, the control substitution S212A showed normal functionality similar to WT TarS. TarP substitution T10P significantly (*P*<0.01) reduced WTA β-GlcNAcylation as well as langerin binding (*P*<0.01) compared to WT TarP (Fig. 5e, f). Lastly, WTA α-GlcNAcylation by TarM was completely abolished by the G17D amino acid substitution (*P*<0.0001) compared to WT TarM (Fig. 5g). In conclusion, we showed that naturally occurring premature stop codons and amino acid substitutions can strongly diminish WTA GlcNAcylation, thereby reducing immune recognition by innate and the adaptive immune components.

## Discussion

GlcNAc decoration of *S. aureus* WTA by glycosyltransferases TarS, TarM and TarP is important for human nasal colonization, β-lactam resistance, phage-mediated horizontal gene transfer and immune recognition [1, 3, 4, 11, 12]. Functional and structural analysis of *S. aureus* Tar-enzymes has been performed using a select number of strains and may not be representative of the entire *S. aureus* population. By analysing 25 652 *S *. *aureus* genomes deposited in the PubMLST database, we confirmed that virtually all *S. aureus* isolates express *tarS* and that 37 and 7 % of isolates co-contain *tarM* and *tarP*, respectively. Co-presence of *tarS/tarM* or *tarS/tarP* correlated with specific *S. aureus* CCs. Moreover, we found a small number of *tar* sequences with natural amino acid substitutions in critical residues of the enzymes or with premature stop codons. By expressing these genes in a *tarMS*-deficient strain, we demonstrated that these genetic variants are severely attenuated in their enzymatic activity *in vivo*, thereby hampering immune recognition of *S. aureus* by innate and adaptive immune molecules.

Previous work on the presence of *tar*-glycosyltransferases only analysed strain collections containing up to ∼100 isolates using PCR [2, 7, 14, 25, 26]. In this study, we analysed 25 652 *S *. *aureus* genomes to obtain a more comprehensive overview of the distribution and genomic variability of the *tar*-glycosyltransferases across the *S. aureus* population. The PubMLST database contained isolates from 19 different CCs of human as well as animal origin, although there is clear skewing towards human clinical isolates. The lack of metadata for many of the deposited genomes does not allow for a complete determination of their origin. Overall, the broad number of species and diversity of CCs renders PubMLST an important tool for *S. aureus* research on the presence and genetic variation of specific genes at the population level.

The presence of *tarP,* which is encoded on three different prophages [7], was previously reported to be restricted to CC5 and CC398^7^. However, we also identified *tarP* in a small number of isolates from CC1, CC7, CC12, CC45, CC59, CC88, CC97 and CC425, and even in *tarM-*associated CC8. This may suggest that *tarP* is present on more, at present unknown, phages, or that the host range of *tarP*-containing phages is broader than currently known.

We found that the presence of *tar* genes as well as sequence (alleles) was associated with specific CCs of *S. aureus *. Most CCs contained a dominant allele for each of the *tar* genes. Since some *S. aureus* CCs display host tropism, these data may suggest that *tar* genes have co-evolved and adapted towards the host. However, as the *tar* genes encode glycosyltransferases that already glycosylate WTA intracellularly, the protein itself is not exposed to the host. Therefore, it may also be that most CCs have a dominant allele for the *tar* genes because of the closely related nature of the isolates belonging to a particular CC. Whether host adaptation plays a role in the presence and sequence of *tar* genes remains to be elucidated.

Overall, our analysis showed that the *tar*-enzymes are highly conserved within the *S. aureus* population and only a small proportion of the analysed genome sequences contained premature stop codons or critical amino acid substitutions. This highlights the importance of WTA glycosylation for *S. aureus *. Indeed, glycosylation of WTA is needed for the horizontal gene transfer of *S. aureus* [3], nasal colonization [1] and β-lactam resistance (independent of *mecA* or other β-lactam resistance-associated genes) [4]. These examples are indicative of why nearly all *S. aureus* isolates contain functional glycosyltransferases, especially *tarS,* and explains the high degree of sequence conservation among the isolates investigated.

Nevertheless, some isolates contained amino acid substitutions in critical residues of the enzymes or premature stop codons, resulting in proteins without or with strongly impaired enzymatic activity. Unfortunately, due to lack of metadata (e.g. provenance, host origin or clinical information) it is hard to determine what kind of host factors and/or genetic mechanisms of *S. aureus* may have contributed to the introduction of these inactivating genetic changes. An overview of the CC distribution (Fig. S4) shows no clear association to a specific ST or CC.

In addition to gene presence and sequence, environmental conditions also affect WTA glycosylation[25]. Indeed, glycosylation by TarM and TarP is dominant over TarS during *in vitro* culture [7, 14]. For TarM this might be explained by a higher inherent enzymatic activity [6], whereas for TarP this may be due to a higher affinity for RboP compared to TarS [7]. Furthermore, *tar* genes may be transcriptionally regulated; *tarM* expression is increased during oxidative stress, probably due to activation of the two-component GraRS regulon [27]. In contrast, *S. aureus* shifts towards TarS glycosylation at the expense of TarM/TarP WTA glycosylation during *in vivo* murine infection models and under high salt conditions [25]. Consequently, the presence of one or more *tar* genes (identified by PCR or genome sequencing) does not provide complete information on WTA glycosylation in a particular isolate. Overall, strain-specific WTA glycosylation will depend on a specific gene sequence in combination with environmentally dependent gene expression and can only be assessed by direct staining methods such as specific Fab fragments.

We investigated the effect of specific genetic mutations on the enzymatic functionality by expressing these variants in a *tarMS*-deficient *S. aureus* strain. This analysis confirmed reduced or abolished activity for several specific amino acid substitutions and premature stop codons in TarS, TarM and TarP. In addition to these naturally occurring amino acid substitutions, we included amino acid substitutions in TarS (D91A, E177A and S212A) that were previously reported to attenuate TarS enzymatic activity *in vitro* [6]. However, in our FACS experiments using live *S. aureus* bacteria, we observed no significant differences in levels of β-glycosylated WTA with the S212A mutation compared to WT TarS, indicating that TarS enzymatic activity was similar to WT TarS. This may suggest that results for TarS enzymatic activity obtained *in vitro*, in which solely enzyme and substrate are present, are not always predictive for WTA β-glycosylation in live bacteria. However, the reason for this discrepancy remains to be elucidated.

The effect of amino acid substitutions and stop codons on WTA glycosylation in live *S. aureus* was assessed with Fab fragments and recombinant FITC-labelled human langerin. Using this system, we could focus solely on the interaction of the antibody or langerin with the WTA-GlcNAc modifications without theF interference of additional molecular interactions as would occur in a cell-based model system. Consequently, it allowed us to more precisely determine the functional consequence of the potentially altered level of WTA glycosylation. Moreover, we have previously shown that langerin engagement by *S. aureus* affects interaction with and downstream responses of langerin-expressing cells such as *in vitro*-generated LCs. Therefore, the experiments using recombinant langerin serve as a proxy for downstream immunological processes in LCs [11, 12]. Finally, it should be noted that we only used the *S. aureus* RN4220 strain, which naturally contains *tarS* and *tarM* [2], and effects may not be identical in other *S. aureus* strains.

In conclusion, *tar* glycosyltransferases are highly conserved and very abundant. In particular, *tarS* was found to be present in >99 % of *S. aureus* strains. We show that there are few exceptions in which *tar* genes seem present but contain amino acid substitutions or premature stop codons, and for these isolates we show that *tar* genotype is not necessarily equal to *tar* phenotype and that thereby immune recognition is hampered. Studying the genetic presence and diversity of *tarS, tarM* and *tarP* provides more insight into *S. aureus* WTA glycosylation, which can help in the development of anti-* S. aureus* preventive or therapeutic interventions such as monoclonal antibodies, phage therapy and vaccines.

## Supplementary Data

Supplementary material 1
