# Supplementary material for: Genetic diversity of Staphylococcus aureus wall teichoic acid glycosyltransferases affects immune recognition
Source: Microb Genom. 2022 Dec 7;8(12):mgen000902. doi: 10.1099/mgen.0.000902 (PMC9837562; doi:10.1099/mgen.0.000902)
Supplement: Supplementary material 1 [file mgen-8-902-s001.pdf]

Genetic diversity of *Staphylococcus aureus* wall teichoic acid glycosyltransferases affects immune recognition

Sara M. Tamminga, Simon L. Völpel, Kim Schipper, Thilo Stehle, Yvonne Pannekoek and Nina M. van Sorge

Supplementary Material

List of materials included:

- Tables S1-S4
- Figures S1-S4

Supplementary Table 1. Bacterial strains used in this study

Strain (sequence type, clonal complex)	Source
RN4220 Δ*tarM*Δ*tarS* (ST8, CC8)	(19)
RN4220 Δ*tarM*Δ*tarS*+pRB474-*tarS*	(19)
RN4220 Δ*tarM*Δ*tarS*+pRB474-*tarM*	(19)
RN4220 Δ*tarM*Δ*tarS*+pRB474-*tarP*	(6)
RN4220 Δ*tarM*Δ*tarS*+pRB474-*tarS*-D91A	This study
RN4220 Δ*tarM*Δ*tarS*+pRB474-*tarS*-D91H	This study
RN4220 Δ*tarM*Δ*tarS*+pRB474-*tarS*-E177A	This study
RN4220 Δ*tarM*Δ*tarS*+pRB474-*tarS*-E177K	This study
RN4220 Δ*tarM*Δ*tarS*+pRB474-*tarS*-S212A	This study
RN4220 Δ*tarM*Δ*tarS*+pRB474-*tarS*-S212R	This study
RN4220 Δ*tarM*Δ*tarS*+pRB474-*tarS*-p124 Stop codon	This study
RN4220 Δ*tarM*Δ*tarS*+pRB474-*tarS*-p625 Stop codon	This study
RN4220 Δ*tarM*Δ*tarS*+pRB474-*tarS*-p892 Stop codon	This study
RN4220 Δ*tarM*Δ*tarS*+pRB474-*tarS*-p1063 Stop codon	This study
RN4220 Δ*tarM*Δ*tarS*+pRB474-*tarM*-G17D	This study
RN4220 Δ*tarM*Δ*tarS*+pRB474-*tarP*-T10P	This study

Supplementary Table 2. Origin of wild-type pRB474 *tar*-gene inserts

Gene on plasmid RB474	Strain of origin	Source	PubMLST allele
tarS	*S. aureus* RN4220	(19)	16
tarM	*S. aureus* RN4220	(19)	1
tarP	*S. aureus* N315	(6)	1

Supplementary Table 3. Primers used in this study

Primer	Target	Sequence (5' to 3')
#018	Plasmid RB474 RV	CTCAAGCTAGAGAGTCATTACCC
#019	Plasmid RB474 FW	CTGGATTTGTTCAGAACGCTCG
#015	TarS RV 1	CTACACGTTCTGGAACTGCTTG
#016	TarS FW 2	GGCATTGATATCGCTAAGCC
#030	TarS FW 1	GTGAACATATGAGTAGTGCGTA
#046	Mutagenesis TarS D91A FW	ATTATTTGTGG**C**CTCCGAT[1]
#047	Mutagenesis TarS D91A RV	CATCGGAG**G**CCACAAA
#040	Mutagenesis TarS D91H FW	GTATTATTTGTG**C**ACTCCGATG
#041	Mutagenesis TarS D91H RV	GTCATCGGAGT**G**CACAAA
#028	Mutagenesis TarS E177K FW	CCAGAAGAATTAAAGAGTGCA**A**AAGATCAATTATTTACAATGAAAGC
#029	Mutagenesis TarS E177K RV	GCTTTCATTGTAAATAATTGATCTT**T**TGCACTCTTTAATTCTTCTGG
#054	TarM RV 1	GCCACTTCAATAAGCAAATC
#053	TarM FW 2	GGGATACCCATATATTTCAAG
#097	TarM FW 4	ATGAAAAAAATATTTATGATGGTACATG
#058	TarP RV 1	GTAGCAAAATATTGATTTCCTG
#057	TarP FW 2	GCGATTTAATAATTGGAAAATATG
#094	pRB474	GTTATTGTCTCATGAGCGGATA
#099	TarP RV	TATCAGCTTTCGCTACATTTCC
#087	gBlock all FW	GCGTTTAAACTT**GGATCC**AAAGGAGGTATTATAATGAT[2]
#088	gBlock S212R/A RV	GATGGGCCCTTGGT**CGTACG**AGAAAAACTAAAATGACGAT
#105	gBlock p124 RV	GATGGGCCCTTGGT**GAATTC**TTATTTATTTTAGCGAGTAA
#106	gBlock p625 RV	GATGGGCCCTTGGT**GAATTC**TTATTTTAGCGAGTAAGTCA
#107	gBlock p892/p1063 RV	GATGGGCCCTTGGT**GAATTC**TTTAGTGGATAAGTGATATG
#100	gBlock TarM G17D FW	GCGTTTAAACTT**GGATCC**AAAGGAGGTATTATAATGAA
#101	gBlock TarM G17D RV	GATGGGCCCTTGGT**GGTACC**ATTCTAGAAATATGACCTACG
#103	gBlock TarP T10P FW	GCGTTTAAACTT**GGATCC**ATGAAAAAAGTAAGTGTTAT
#104	gBlock TarP T10P RV	GATGGGCCCTTGGT**TTCGAA**TTCATCAAAAATTCTATAGT
#109	TarS-p124 FW	TGAAAGGTGTCAATGGTCGTGG
#110	TarS-p124 RV	CGTTGATTACCAACACGCACTTC
#111	TarS-p625 FW	GAATGCAAATCGAATCAGTGTG
#112	TarS-p1063 FW	GATTGGCGAAGGACATTATGAT

[1]All nucleotide substitutions are indicated in bold. [2]Restriction digestion sites are underlined.

Supplementary Table 4. Number (%) of *tar*-enzymes present in different clonal complexes

CC	*tarS*	*tarM*	*tarP*
CC1	1,030 (4.0%)	424 (4.5%)	49 (2.7%)
CC5	5,761 (22.6%)		1,205 (67.3%)
CC7	49 (0.2%)		2 (0.1%)
CC8	6,661 (26.2%)	6,600 (69.5%)	15 (0.8%)
CC12	60 (0.2%)	2 (0.02%)	2 (0.1%)
CC15	436 (1.7%)	398 (4.2%)	3 (0.2%)
CC22	5,093 (20.0%)		
CC25	96 (0.4%)		
CC30	1,383 (5.3%)	1,351 (14.2%)	3 (0.2%)
CC45	584 (2.3%)		38 (2.1%)
CC59	154 (0.6%)		2 (0.1%)
CC88	111 (0.4%)		5 (0.3%)
CC93	446 (1.8%)	446 (4.7%)	
CC97	300 (1.2%)		12 (0.7%)
CC121	132 (0.5%)	108 (1.1%)	
CC130	465 (1.8%)		
CC133	247 (1.0%)		
CC398	1,347 (5.3%)		397 (22.2)
CC425	141 (0.6%)		2 (0.1%)
Unknown	970 (3.8%)	162 (1.7%)	56 (3.1%)
**Total**	**25,466 (100%)**	**9,491 (100%)**	**1,791 (100%)**

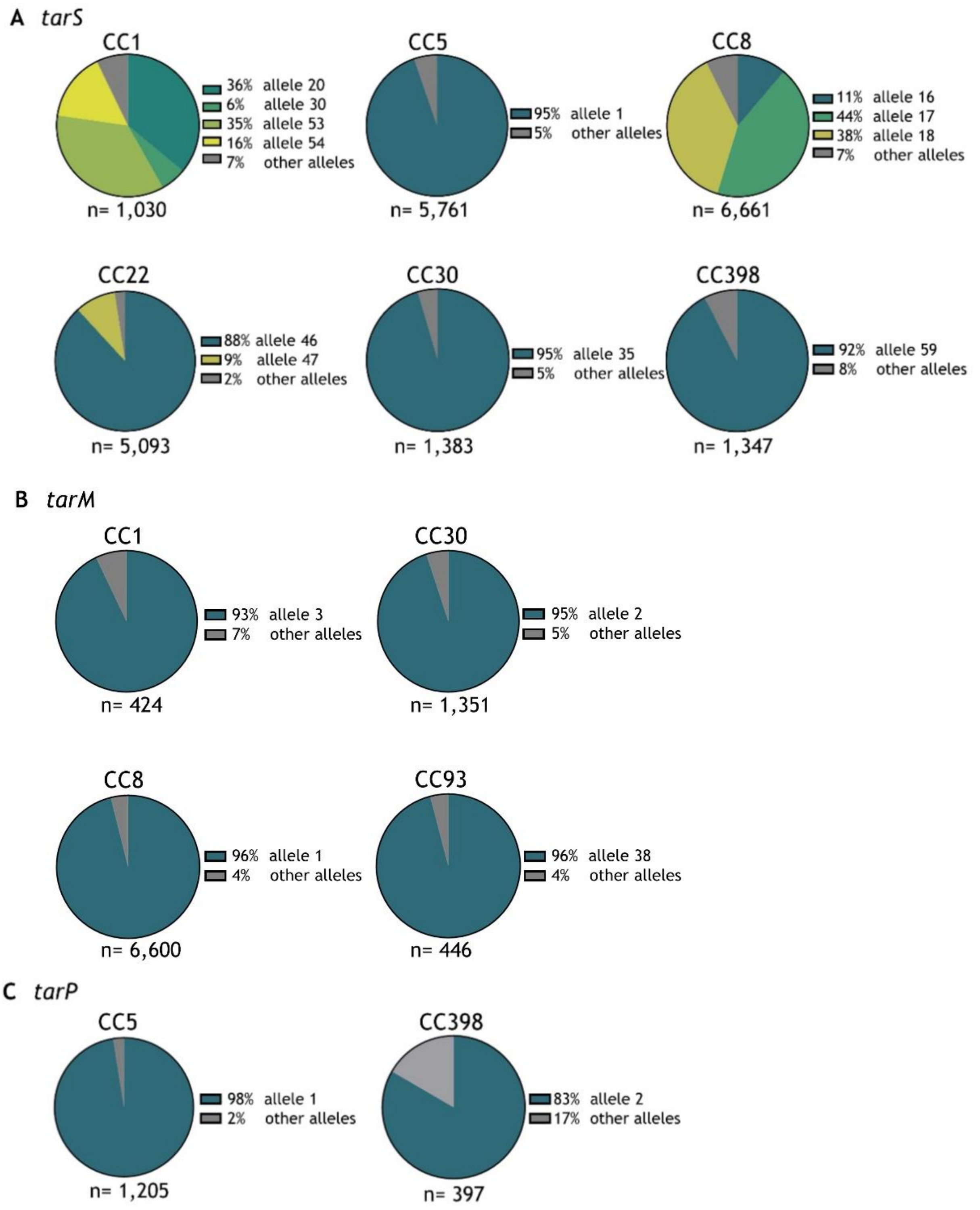

Supplementary Figure 1. Distribution and occurrence of *tar* alleles within different clonal complexes. (A) The most frequently observed *tarS* alleles in distinct clonal complexes (CCs that comprise >4% of total isolates are depicted). (B) Same as in (A) but for *tarM.* (C) Same as in (A) but for *tarP.*

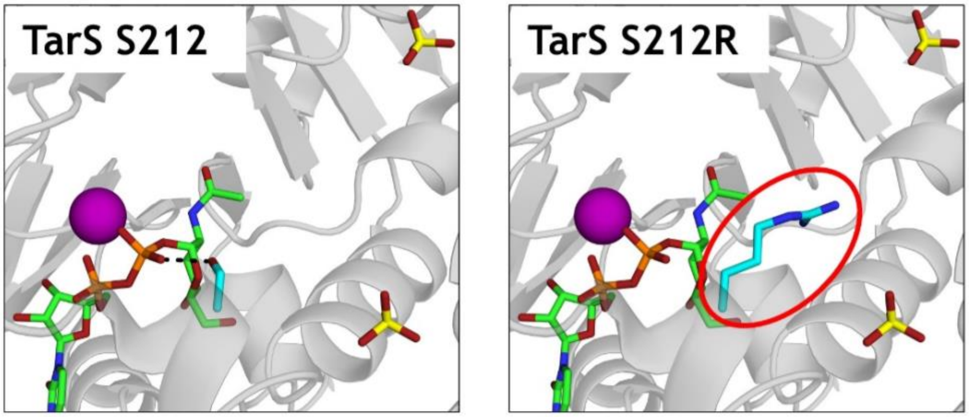

Supplementary Figure 2. PyMOL visualizations of TarS S212 and S212R. TarS is shown in grey cartoon presentation (PDB code 5TZE). The donor substrate UDP-GlcNAc (green) and the residues of interest: S212 (left) and the substitution S212R (right, red circled), both in cyan, are all displayed in stick form. Sulfates are indicated in yellow.

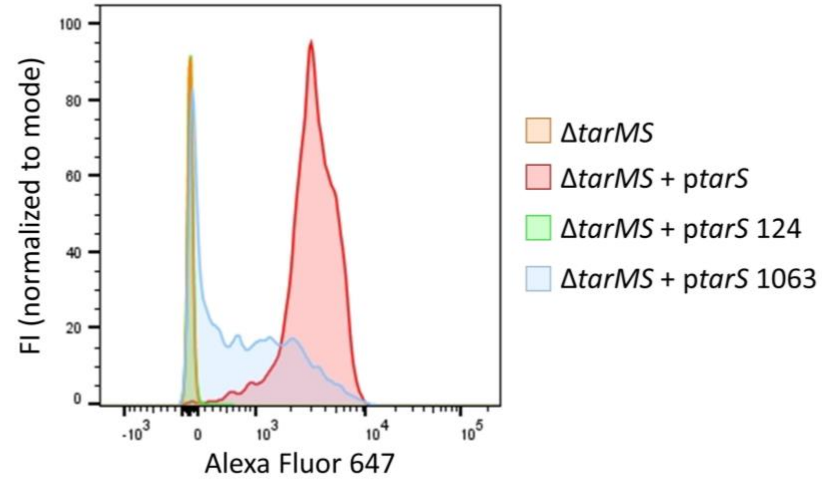

Supplementary Figure 3. Representative flow cytometry histogram. Raw flow cytometry data corresponding to Figure 5A. Binding of monoclonal Fab fragments specific to β-GlcNAc-WTA (4497) to *S. aureus* RN4220 Δ*tarMS* complemented with plasmid-expressed WT *tarS* or premature stop codon 124 or 1063. Histogram of fluorescent intensity (FI) normalized to mode for secondary antibody (Goat $F(ab')_2$ anti-human Kappa-Alexa Fluor 647) is shown.

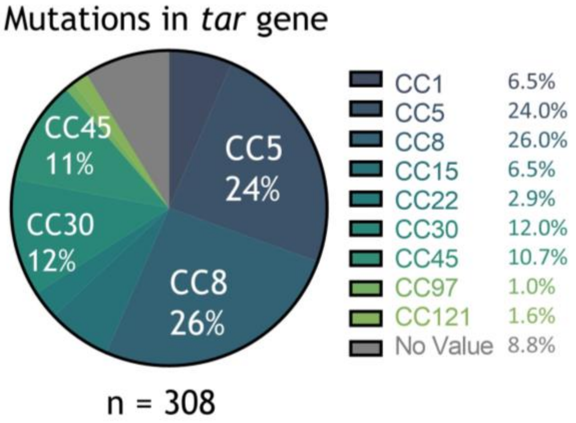

Supplementary Figure 4. Overview of CC distribution of isolates that contain an amino acid substitution or a premature stopcodon in a *tar* gene. CC distribution of the 308 isolates that were identified containing an amino acid substitution in a critical residue or a premature stopcodon in one of the *tar* enzymes.
